# Environmental manipulation for edible insect procurement: a historical perspective

**DOI:** 10.1186/1746-4269-8-3

**Published:** 2012-01-21

**Authors:** Joost Van Itterbeeck, Arnold van Huis

**Affiliations:** 1 Laboratory of Entomology, Department of Plant Sciences, Wageningen University, Droevendaalsesteeg 1, 6708 PB Wageningen, the Netherlands

**Keywords:** edible insect, entomophagy, facilitation, environmental manipulation, semi-cultivation, aquatic insect egg, *ahuauhtle*, palm weevil, palm larvae, caterpillar

## Abstract

Throughout history humans have manipulated their natural environment for an increased predictability and availability of plant and animal resources. Research on prehistoric diets increasingly includes small game, but edible insects receive minimal attention. Using the anthropological and archaeological literature we show and hypothesize about the existence of such environmental manipulations related to the procurement of edible insects. As examples we use eggs of aquatic Hemiptera in Mexico which are semi-cultivated by water management and by providing egg laying sites; palm weevil larvae in the Amazon Basin, tropical Africa, and New Guinea of which the collection is facilitated by manipulating host tree distribution and abundance and which are semi-cultivated by deliberately cutting palm trees at a chosen time at a chosen location; and arboreal, foliage consuming caterpillars in sub-Saharan Africa for which the collection is facilitated by manipulating host tree distribution and abundance, shifting cultivation, fire regimes, host tree preservation, and manually introducing caterpillars to a designated area. These manipulations improve insect exploitation by increasing their predictability and availability, and most likely have an ancient origin.

## Introduction

Much research on prehistoric diets focused on the procurement of meat, i.e. hunting of large animals. This has been considered to be an important socio-cultural aspect in antiquity. McGrew [1] warns for a skewed picture of early diets. Regarding animal protein, archaeological evidence throughout the world points to a broad diet that included fish, birds, lizards, rodents, rabbits, turtles, crabs, molluscs, and shellfish [2]. Coprolite analyses provide direct evidence also of the consumption of insects e.g. termites and predaceous diving beetles by early American Indians (up to 9,500 B.P.) and ants, dung beetle larvae, and caterpillars in Mexico (> 5,400 B.P.) [[3] and references therein, see also [4]].

For an understanding of prehistoric life, archaeologists increasingly consult anthropological studies of traditional forager societies today, such as the !Kun-San of the Kalahari and Australian Aborigines, which are believed to resemble such life most closely [2,5]. It is increasingly acknowledged that studies on the ecological anthropology of these peoples have usually wrongfully ignored the use of insects as food. The !Kun-San consume termites, grasshoppers, caterpillars, and ants [6]. Australian Aborigines consume e.g. termites, grasshoppers, moths, caterpillars, beetle larvae, wasp larvae, and ant brood [7-9]. Their practice of entomophagy has decreased though over the past 200 years through western influence [10].

Some other studies incorporating insect use were conducted in the Amazon Basin [11-13]. The indigenous groups under investigation practice forms of cultivation and horticulture in combination with hunting and gathering. Such combinations in subsistence have been practiced throughout history [2]. These records indicate intensive use of insects as food source. Based on such archaeological and ethnographic records, Sutton [4] stresses that "... we must *expect *[original italics] the use of insects in antiquity and so we must subject insect remains to the same examination and analysis that we do for other small animals".

From the perspective of hunting *individual *animals in relation to their body size, insects can generally be considered low ranked food sources as the return rates (energy gained minus energy costs from searching, handling, and processing) of large animals is higher [14]. Madsen and Schmitt [14] reason that an increased abundance in such lower ranked resources (e.g. in patches, clumps, and swarms where the collected/hunted unit is no longer an individual) and/or the introduction of mass collecting technology increases their return rates to an equally high or higher level than that of large animals collected individually.

This theory is based on evidence from Lakeside Cave (Great Salt Lake, Utah, USA) where excavations and analyses of faecal remains covering the past 5,000 years indicate a switch from mammal to locust consumption when abundant [14]. Following outbreaks, vast numbers of locusts (e.g. *Melanoplus sanguinipes*) drowned in the lake and washed up on the shores in windrows numerating to tens-of-thousands, making collection easy. The lake served as a natural collecting mechanism enabling mass collection. The return rates of locusts increased, at least in the direct vicinity of the lake, even with unchanged abundance in large animals. This caused a shift in focus from large, high ranked animals to small, normally low ranked animals [14]. Such locust outbreaks were no annual phenomena, but occurred twice every decade since the 1850s. They have been relatively common yet unpredictable [15].

In an anthropological study of indigenous populations Steward [in 16] indicates though that in this area small game, i.e. rodents, lizards, and insects, probably already contributed more to the diet than large animals due to the latter's scarcity and lack of occurrence in herds. Families exchanged useful information on locations of those resources including locust concentrations.

Seemingly in line with Madsen and Schmitt's [14] findings and theory, the Bogong moth (*Agrotis infusa*) was the most reliable summer food source for some Australian Aborigine tribes and preferred over other food sources available at the same time. As Bogong moths naturally congregate by the masses in crevices in rocks and in recesses between rocks in the Australian Alps, large quantities could easily be collected [17]. The traditional Aborigine diet was furthermore overall low in fat [7,18,9] and fat-rich grubs and moths such as the Bogong moth may have been important nutritionally [17]. These moths also played a role in socio-cultural aspects: ceremonial life, marriage, and trade. Their collectors are now referred to as moth hunters [17].

McGrew [1] points to such importance of energetic efficiency and suggests that a large-brained forager could be able to deal with this issue by means of intelligent strategies making the gathering/collecting of animal protein as productive as the strategies of hunting. Such strategies may have very well included, as it was for various other resources [2], besides the construction of tools, the manipulation of the environment related to insect procurement [1,19].

This invites us to review findings of facilitating edible insect procurement beyond the construction of tools and reinterpret environmental manipulation reported in the archaeological and anthropological literature. As examples we use the following semi-cultivated edible insects: eggs of aquatic Hemiptera in Mexico, palm weevil larvae in the Amazon Basin, tropical Africa, and New Guinea, and arboreal, foliage consuming caterpillars in sub-Saharan Africa.

The prime source of literature was the entomophagy bibliography of the authors containing more than 1500 publications (the majority peer reviewed) of which more than 600 publications deal specifically with edible insects and the human practice of entomophagy - eating insects. The aforementioned examples were chosen based on the amount of information available. Additional information on these examples was collected via internet search engines. This review excludes edible insects that are primarily semi-cultivated or domesticated for their products such as bees, wasps, and silkworms.

## Water management for eggs of aquatic Hemiptera in Mexico

When comparing food procurement of ancient civilizations it is remarkable that only pre-Hispanic Mesoamerica achieved organizational complexity and high population density without a domesticated herbivore. Non-agricultural high-protein resources are expected to have been used intensively [20]. These are particularly aquatic fauna and flora of which the eggs of aquatic true bugs (Hemiptera: Neopomorpha) (*ahuauhtle*) are regarded of specific importance [20]. A delicacy among the Aztecs, the eggs were called 'Mexican caviar' by the Spanish conquistadores [21].

The ponds and marshes of the valleys and basins of the Mexican Mesa Central were of such importance to subsistence, energetically, nutritionally, and economically, that their contribution holds comparison with agriculture. Fish and birds were caught, algae collected, and insects netted. These activities were conducted by large numbers of people [20]. The *ahuauhtle *(the adults are called *axayacatl*), measuring about 0.5 to 1.0 mm, are the eggs of particularly *Krizousacorixa *spp., *Corisella *spp., and *Corixa *spp. (Hemiptera: Nepomorpha: Corixidae) and *Notonecta *spp. (Hemiptera: Nepomorpha: Notonectidae) [20-22]. They are deposited on aquatic vegetation throughout the year [20]; according to Bachstez and Aragon [21] from June to October in Lake Texcoco (located in that same area). Insects and their products (and algae) were collected in higher amounts than other aquatic resources while their nutritional value exceeds that of those other food resources. Parsons [20] roughly estimates an insect harvest of 10 kg and insect egg harvest of 5 kg every two weeks per ha reaching an annual insect/insect-egg harvest of 3,900 metric tonnes for an assumed available lake surface area of 10,000 ha. This is an impressive figure in regard of the nutritional value: insects are generally good sources of protein, minerals, vitamins, fibre, fatty acids, and essential amino acids [23] and *ahuauhtle *(eggs) contains 0.20% Ca, 0.33% Fe, 5.7% fat, 77% protein, and 0.73% phosphor [21]. Bergier [22] claims from examining samples from the 1850s that only the egg shells would have been collected. That would be rather unlikely although it is certainly possible that part of the eggs had already hatched.

The intensive exploitation of *ahuauhtle *is facilitated, referred to as semi-cultivation [20,24]. The techniques employed reflect indigenous knowledge of the oviposition behaviour of aquatic Hemiptera. Locals made bundles of twigs of grasses or reeds, e.g. *Carex *[25], bound them together with a rope, and placed many of these bundles on the bottom of the lakes at certain distances from each other. To keep them in place, a stone was tied to the rope or the bundles were simply pushed in the lake bottom [20,25,26]. More recently, long U-shaped grass/reed bundles were placed at one meter intervals. The female Hemiptera lay their eggs on these bundles which can be harvested shortly after. This facilitation works best in still and shallow water which requires skills in engineering and management practices of the lakes, skills that may well have been present in ancient times [20,27]. A map of Lake Texcoco from 1550 suggests a division of activities to two sides of the lake divided by, possibly, a reed barrier. The nature of the activities depicted suggests that the shallow side was used for insect farming, as it was more suitable for netting insects and exploiting eggs of aquatic Hemiptera [20].

## Host plant manipulation for palm weevil larvae in the Amazon Basin, tropical Africa, and New Guinea

The larvae of several species of palm weevils (Coleoptera: Curculionidae) are edible including the Asian *Rhynchophorus ferrugineus *[28] and *R. bilineatus *[29], the African *R. phoenicis *[30-32], and the American *R. palmarum*, *Rhinostomus barbirostris*, *Dynamis borassi *and *Metamasius *sp. [33]. While the soft-bodied larvae are favoured, Townsend [34] reports that adults may also be eaten. Facilitation of palm weevil larvae procurement, also referred to as semi-cultivation, is practiced by various indigenous groups and well reported in Asia [28,34,35], Africa [36], and South America [11,13,33,37-39]. The detailed work of Choo et al. [39] is highlighted here who report that based on their Traditional Ecological Knowledge of palm weevils (*Rhynchophorus palmarum *and *Rhinostomus barbirostris*) the Jotï can exercise controlled supply by deliberately felling palm trees for harvesting the larvae.

Aforementioned weevils differ in their ovipositing biology. Whereas *R. palmarum *adults are attracted to exposed inner palm tissue of felled or naturally fallen palms where they feed, mate, and oviposit, *Rh. barbirostris *females oviposit on the intact surface of the trunk and are thus able to use the entire trunk length for oviposition. When harvesting 1 - 3 months later, larvae of *Rh. barbirostris *are then more abundant than those of *R. palmarum*. With the flavour and fat content of these weevil larvae differing, the Jotï, depending on personal preferences, manipulate species abundance in favour of *R. palmarum *which arrives at the trunks earlier than *Rh. barbirostris*. This requires an increased availability of softer inner tissue which is provided by making deep cuts in the trunk [39]. Paraguayan Indians cut the trunk in smaller pieces for the same goal [40] while the Korowai of Western Papua make holes in the trunk to ease weevil access [41]. Palm weevil mating behaviour is gregarious which easily results in 100 larvae and more in a single trunk. As adult weevils only oviposit in unworked portions of trees that are cut to harvest starch [42], deliberately felling trees for harvesting of larvae would thus increase their numbers.

A variety of palm trees is used but in the Amazon Basin the *moriche *palm (*Mauritzia flexuosa*) renders highest larval density [33]. In Papua New Guinea sago palm species (*Metroxylon *spp.) that render lower amounts of starch are rather felled for palm weevil larvae (*Rhynchophorus ferrugineus *and *R. bilineatus *[43]) semi-cultivation presumably for the matter of energetic efficiency [34].

The products provided by palm trees, e.g. fruit, starch, building material, and palm weevil larvae, are of general importance to many indigenous groups, and were of importance in prehistory [2,41,44]. Manipulation of the forest, and palm tree distribution and abundance specifically, has occurred throughout history [2]. The Nukak are a South American hunting and gathering group that seem to manage or manipulate various plant species including palm trees. Politis [45] suggests that this observation of manipulation is due to the Nukak being a mobile group that frequent previous camp sites. High concentrations of seeds are left each time thus favouring those species. In a similar way Hurtado et al. [46] found palm trees to occur in a somewhat uniform distribution in the forest inhabited by Aché Indians. Explanations of such manipulation are primarily in light of starch which may be a staple. Though the larvae are an acclaimed delicacy for which even large feasts are held and in preparation of it numerous trees are deliberately felled, they are considered a by-product by Abd-Aziz [41]. Dufour [13] refers to the larvae as a by-product when the Tatuyo Indians fell trees for harvesting the fruits, and refers to them as "cultivated" only when trees are felled specifically for palm weevil larvae facilitation. In Africa, *R. phoenicis *larvae are harvested from trunks that have been cut down to extract sap which is used for making palm wine. Adult weevils are attracted to the rotting trunk and invade it [36,47]. Since after harvesting the fruits or extracting the sap the trunks are left and visited at a later date to harvest the larvae, it is possible that a dual production of both fruits and larvae is intended [34]. Bodenheimer [48] therefore refers to the larvae as "a second crop". Johnson [49] made an assessment of various palm species in terms of the number of products they provide (multipurpose palms) but did not include palm weevil larvae. More careful consideration is required when classifying weevil larvae as a by-product. We thus argue that the role of these larvae in palm tree distribution and abundance manipulation may be underestimated.

Archaeological evidence suggests that the strategies and technologies observed today could very well be similar to those practiced several thousands of years ago, making rainforest exploitation more efficient and effective [2]. As described, the Jotï fell palm trees deliberately, and they do so at a chosen location at a chosen time thereby controlling the place and time for harvesting the larvae [39]. The Yapú employ this as a strategy in long hunting and fishing trips that lead them away from their village [13]. By felling palm trees beforehand the weevil larvae are a more predictable resource than by natural collection as search time is decreased [39]. Moreover, during periods of game and fish scarcity, the larvae are expected to become a more important source of protein and fat [12,23,50]. In both cases, the availability of concentrated patches and/or a uniform distribution of palm trees, as seems to occur among the Nukak [45] and Aché Indians [46] respectively, would then 'facilitate the facilitation' of the palm weevil larvae harvest. Indications exist in the archaeological evidence of the management and protection of at least sago palms while interventions such as forest clearance, planting, and plant-tending were common throughout history [2].

## Host plant manipulation for arboreal, foliage consuming caterpillars in sub-Saharan Africa

The far majority of edible caterpillars (Lepidoptera) reported in sub-Saharan Africa are leaf feeders [51,52]. Latham [53] reports from the Bas Congo the introduction of caterpillars *Imbrasia obscura*, *Imbrasia ertli*, and *Cirina forda *in selected areas. People collect or buy young caterpillars and place them in trees near their home and on their land. Similarly, Malaisse [30] mentions the translocation of a branch with many caterpillars to the same tree species closer to one's village. When the caterpillars reach maturity (final larval stage and largest size) they are collected to eat [53]. In this system people can keep an eye on their caterpillars thus improve the timing of collection: only those caterpillars large enough will be taken. Furthermore, some of the caterpillars are allowed to pupate below the trees [53]. Provided that during this stage the area is protected from fire, the resulting moths of certain species will lay eggs on the tree below which they pupated or on nearby trees [53]. A farmer followed this system of facilitation by introducing *Cirina forda *caterpillars to an area of savannah abundant in the tree species *Crossopteryx febrifuga *and could harvest caterpillars on a regular basis since this introduction [53].

Silow [51] mentions a traditional tendency to domesticate caterpillars but does not provide details. Though, he does explain that those caterpillars that feed on more than one host tree differ in flavour and thus may be translocated by epicures from one host tree to another to improve the caterpillars' flavour. It would indeed be most convenient to translocate them closer to home.

Four forms of traditional facilitation through habitat management, all of which increase caterpillar yields, whether or not employed in combination, are reported. First, host tree planting in predetermined areas attracts moths [54] which oviposit eggs on the foliage. Second, shifting cultivation stimulates re-growth of caterpillar host trees. Re-growth of miombo woodland (*Brachystegia-Julbernardia*) on land previously under cultivation in Zambia is shown to be dominated by *Julbernardia paniculata *(the main host tree of e.g. *Gonimbrasia *(*Imbrasia*) *zambesina *and *Gynanisa maja *caterpillars) in comparison to old-growth woodland such as forest reserves [55]. Third, applying correct fire regimes protects host trees, and avoids destruction of moth eggs on foliage and the pupae underground [56-58]. Fourth, not cutting down host trees preserves caterpillar 'breeding sites' [59].

In addition, traditional regulations may involve the monitoring of caterpillar development and abundance. Mbata et al. [58] provide a detailed account of such activities on caterpillars (e.g. *Gynanisa maja *and *Gonimbrasia zambesina*) in the Kopa area in Zambia which include the identification of high density moth egg sites, the appearance of first instar larvae, and the appearance of final instar larvae (the only instar allowed to be collected). The monitoring is well organised and continuous throughout the season whereby all levels of society are involved, rituals are performed, and ceremonies held. When necessary, the observations result in temporal restrictions in harvesting [58].

Caterpillars are a widely consumed resource, particularly in Africa, with over 40 edible species reported for Congo DR alone [53,60]. They are a popular food, harvested in large quantities by numerous people, and in lean times are of specific importance. The facilitation techniques employed are straightforward and stem from indigenous knowledge of caterpillar biology [e.g. 58]. These types of strategies - plant manipulation, use of fire - have been employed in antiquity throughout the world [2]. It seems highly unlikely that forms of landscape management in support of caterpillar exploitation is a recent development.

## Discussion

### Revaluating the importance of insects as food in history

Combining archaeological evidence from coprolites and excavations with ethnographic studies of indigenous peoples suggests human entomophagy - eating insects - to be an ancient habit [1,4]. A large variety of plants and animals were manipulated in antiquity [2], but edible insects have so far received little attention concerning this matter. Barker [2] points in his extended review to zeitgeist, methodologies applied, and techniques available: "... the questions being asked [by an archaeologist] affect the kind of methodologies used in the field, prioritizing the recovery of certain classes of information over others. New theories will pose new questions about past societies, and these questions will result not just in reinterpretations of existing data but also in new kinds of data being collected in the field or the laboratory. New data, whether searched for explicitly or thrown up by unexpected discoveries, feed back into theoretical frameworks."

The general Western cultural and historical bias towards male activities and hunting of large game is gradually dissolving, the data providing a fuller picture of the various modes of subsistence. Particularly Sutton [4,5,19] has previously made a case for (edible) insects, arguing the bias against this one category of 'small game'. The retrieval, identification, and interpretation of insect remains is problematic, Sutton [4] clarifies practical archaeological issues. The reinterpretations made here are problematic, particularly for the cases of palm weevil larvae and caterpillars, as it is difficult, if not impossible, to provide direct evidence of the manipulations to be also insect related. Thus the majority of hypotheses concerning such landscape manipulations must come forth from reinterpretations fed by anthropological findings of insect use, and if available, supported by ancient scripture (as in the case of *ahuauhtle*) and ancient depictions (such as Egyptian hieroglyphs of honey extraction). The cases described here can contribute to new archaeological theories on insect use and its role, leading to reinterpretations of existing evidence (e.g. fire, tree distribution, tools), the collection of new data, and a feedback into theoretical frameworks.

### The path to domestication

It was not a sudden event that transformed foragers into farmers, nor was it a discovery or invention, but a long process, a transition, spanning several thousands of years. Landscape manipulations of the kind described above are steppingstones in the development of domestication/agriculture and have their origin most likely in a need to increase resource predictability and availability [2].

The development of domestication/agriculture is considered a revolution with regard to the effect it had on human societies [2] though it has its negative effects as well e.g. through rapid population growth [61]. DeFoliart [62] states: "Considering their efficiency in converting plant biomass to animal biomass, the failure to domesticate edible insects on any significant scale (except as a by-product of silk and honey production [and carmine production for e.g. cloth and food colouring]) may have been a greater calamity in the global development of agriculture than we yet realize." Research is and has been carried out to semi-cultivate and domesticate edible insects and to optimize existing techniques, e.g.: bamboo worm (*Omphisa fuscidentalis*) [63], Mopane worm (*Imbrasia belina*) [64,65], termite (Isoptera) [66,67], palm weevil larvae (e.g. *Rhynchophorus palmarum *and *R. ferrugineus*) [[33], Yupa Hanboonsong personal communication], cricket (*Acheta domesticus*) [[68], Yupa Hanboonsong personal communication], and weaver ant (*Oecophylla smaragdina*) [69,70]. In fact, weaver ant nests (Hymenoptera: Formicidae: *Oecophylla smaragdina*), made by worker ants by 'weaving' together living leaves with larval silk [71], are traditionally taken from the forest and placed in trees in gardens and plantations in Thailand as their brood is a valued source of food [[68], Yupa Hanboonsong personal communication]. As weaver ants nest in a large variety of trees [72] environmental manipulations in the sense described above are however less likely to have occurred in history.

Some scholars advocate an increased use of small game (mini-livestock) [73-76]. Breeding edible insects has for example environmental advantages over breeding conventional livestock such as their efficiency in converting feed into biomass [77] and a lower greenhouse gas production [78]. Research is showing positive results yet issues remain to be tackled to assure successful edible insect production e.g. susceptibility to viral and bacterial diseases [64,79]. Perhaps in the beginning of next century a scholar will review these developments and adapt Graeme Barker's [2] title: *The Agricultural Revolution in the 21^st ^Century - Why did livestock farmers become mini-livestock farmers?*

## Conclusions

Three examples were presented here that show and hypothesize about manipulations of the environment related to edible insect procurement. The exploitation of eggs of aquatic Hemiptera in Mexico, palm weevil larvae in the Amazon Basin, tropical Africa, and New Guinea, and arboreal, foliage feeding caterpillars in sub-Saharan Africa is facilitated, enhancing the predictability and availability of these edible insects, up to a form of semi-cultivation. These manipulations allow for large numbers of small animals to be collected efficiently, thereby increasing return rates. The facilitation of harvesting the edible insects dealt with in this paper can be regarded as an intelligent strategy for the procurement of, particularly, animal protein and fat. However limited the evidence presented here may be, the likeliness of insect related manipulations of the environment both in the present and the past is not that farfetched, even when it concerns but few species and insects not necessarily being the only or main reason for the manipulations.

## Competing interests

The authors declare that they have no competing interests.

## Authors' contributions

JVI reviewed the literature and drafted the manuscript. AvH critically revised the manuscript and gave final approval of the version to be published. All authors read and approved the manuscript.
